# Alternative Splicing Isoforms of Porcine *CREB* Are Differentially Involved in Transcriptional Transactivation

**DOI:** 10.3390/genes13081304

**Published:** 2022-07-22

**Authors:** Dongjie Zhang, Qian Zhang, Liang Wang, Jiaxin Li, Wanjun Hao, Yuanlu Sun, Di Liu, Xiuqin Yang

**Affiliations:** 1 Institute of Animal Husbandry, Heilongjiang Academy of Agricultural Sciences, Harbin 150086, China; djzhang8109@163.com (D.Z.); wlwl1448@163.com (L.W.); 2 College of Animal Science and Technology, Northeast Agricultural University, Harbin 150030, China; 15169615521@163.com (Q.Z.); ljxneau@163.com (J.L.); haowanjun1109@163.com (W.H.); sunyuanlu2021@163.com (Y.S.)

**Keywords:** pig, CREB, alternative splicing variants, function, RNA-seq

## Abstract

The cAMP response element-binding protein (CREB), a basic leucine zipper transcription factor, is involved in the activation of numerous genes in a variety of cell types. The *CREB* gene is rich in alternative splicing (AS) events. However, studies on the AS of *CREB* genes in pigs are limited, and few reports have compared the roles of isoforms in activating gene expression. Here, five AS transcripts, V1–5, were characterized by RT-PCR and two, V3 and V5, were new identifications. Both V1 and V2 have all the functional domains of the CREB protein, with similar tissue expression profiles and mRNA stability, suggesting that they have similar roles. The transcriptional transactivation activities of four isoforms encoding complete polypeptides were analyzed on the expression of the B-cell CLL/lymphoma 2-like protein 2 and the poly (A)-binding protein, nuclear 1 genes with a dual-luciferase reporter system, and differential activities were observed. Both V1 and V2 have promoting effects, but their roles are gene-specific. V3 has no effect on the promoter of the two genes, while V4 functions as a repressor. The mechanisms underlying the differential roles of V1 and V2 were analyzed with RNA-seq, and the genes specifically regulated by V1 and V2 were identified. These results will contribute to further revealing the role of CREB and to analyzing the significance of AS in genes.

## 1. Introduction

The cAMP response element (CRE)-binding protein (CREB), also known as CREB1, is a 43-kDa basic leucine zipper (bZIP) transcription factor (TF). It is involved in the activation of many cellular signals through the regulation of genes harboring the CRE motif. Genome-wide screening for CRE motifs indicated that more than 4000 genes might be activated by CREB. This suggests that CREB is a general TF and is important in many cellular processes [1]. *CREB* gene knockout mice had embryonal neuronal deficits and a reduced lifespan [2,3]. The dysregulation of the *CREB* gene results in aberrant signal transduction affecting apoptosis, proliferation and differentiation, immune surveillance, and metabolism [4,5,6,7].

The consensus sequence of the CRE motif is TGACGTCA, and it was first characterized in the neuropeptide somatostatin gene [8]. The CREB gene also recognizes the conserved half (TGACG or CGTCA) of the CRE sequence. The CGTCA sequence in the promoter of TAT or Sox 3 has been identified as a prototypical binding site of CREB [9,10,11]. Many genes containing TGACG are targets of CREB, including c-Fos [10], CYP11B [12], and Sox 9 [13]. A few nucleotide variations from the CRE consensus sequence in some genes have little effect on the binding of CREB, except for affinity changes [14,15,16]. Some genes with a nonpalindromic CRE motif, such as the half CRE element, TGAGC, are more efficient in the induction of CREB than the full ones [14]. This might be because the competitive TFs, such as ATF2 or ATF3, for CRE cannot bind to the half CRE element [17], or because the affinity is attenuated [18] and the binding of CREB is easier on the half sequences.

There are many alternative transcript variants encoding distinct polypeptides in the *CREB* gene [19,20,21]. Some rare isoforms identified in the testis have been implicated in spermatogenesis in rats [22] and humans [21]. This indicates that the alternative splicing (AS) of the *CREB* gene has functional significance and physiological importance. Among the CREB alternative protein isoforms, isoforms A (NM_004379) and B (NM_134442) are major forms and are universally expressed in tissues. Both of these isoforms contain all the functional domains of CREB, including the N-terminal glutamine-rich (Q) domains, the kinase-induced domain (KID), and the C-terminal bZIP domain that have similar expression profiles in mice [19]. It is unlikely that two isoforms with identical roles exist simultaneously in tissues. However, it is not well established whether these isoforms are different in their transcriptional transactivation potential and whether the specific signals regulated by each isoform exist. In this study, we aimed to analyze the differential roles of the CREB isoforms and characterize novel AS transcripts in pigs.

CREB plays important roles in many physiological and pathological processes in humans [23,24]. Pigs have many histological and physiological similarities to humans, and they have been used as an animal model for many human diseases. Our results will help to reveal the detailed roles of CREB and contribute to the improvement of human health. Additionally, pork accounts for more than 50% of the total meat consumption in China. The clarification of the roles of CREB, a general TF that is important for cellular processes, might help to improve the growth and productivity of pigs.

## 2. Results

### 2.1. Identification of Alternative Splicing Transcripts

A total of five AS variants (named V1–5) were obtained by RT-PCR from mixed RNAs isolated from the spleen, kidneys, muscles, lungs, stomach, liver, heart, fat, large intestine, and small intestine of pigs. V1, V2, and V4 have the same coding sequence (CDS) as those deposited in GenBank (NM_001361427, NM_001099929, and XM_021075309) and encode the polypeptides of 341, 327, and 278 aa, respectively. V1 and V2 correspond to isoforms B (NM_134442) and A (NM_004379) in humans [21,25], respectively. Both V1 and V2 show a similarity of 100% with the homologs from humans. Transcripts V3 and V5, encoding the polypeptides of 299 and 104 aa, respectively, were identified for the first time.

**Figure 1:**
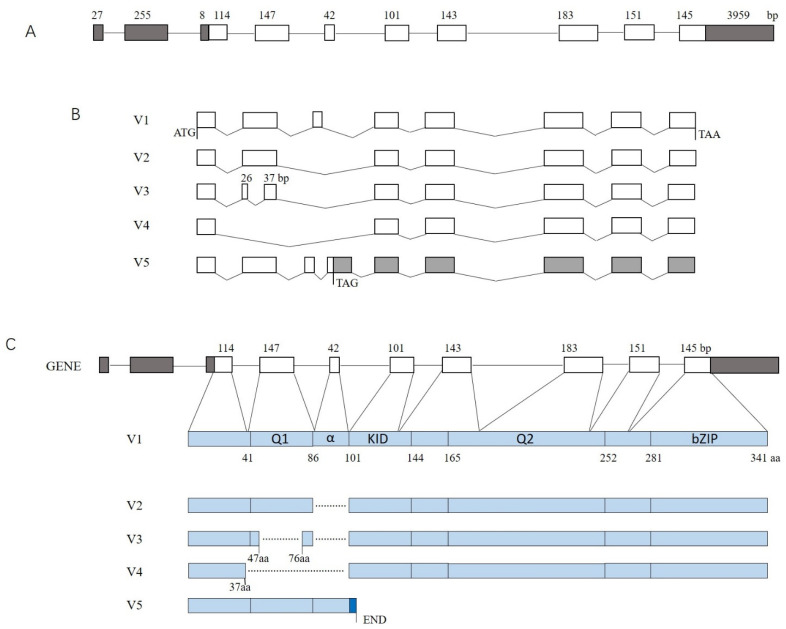
Schematic structure of the identified transcript variants of the porcine *CREB* gene. (**A**) Schematic diagram of the *CREB* gene (NM_001361427) structure. (**B**) Schematic structure of the alternative splicing transcripts. (**C**) Domain composition of the alternative splicing isoforms. Exons and introns are indicated with a box and a straight line, respectively. Oblique lines indicate that the sequences are spliced out. A blank box indicates a coding sequence, while a closed box indicates an untranslated region.

The porcine *CREB* gene is composed of 10 exons, and the CDS starts from the ninth base pair of exon 3 (Figure 1A). All the splice sites in transcript V1 follow the GT-AG rule. In contrast with V1, V2–4 are formed by in-frame deletions. Both V2 and V4 are formed by exon skipping. V2 is absent from exon 5, and V4 lacks exons 4 and 5. V3 is formed by an internal deletion of 84 bp in exon 4, spanning the sequence from 27 bp to 110 bp, indicating the presence of a cryptic intron. The splice sites of this cryptic intron are AT-CC. V5 contains a premature termination codon (PTC) owing to the retention of a partial sequence of intron 5 and encodes a truncated polypeptide (Figure 1B). Q1, KID, Q2, and bZIP domains exist in sequence in the polypeptide of variant V1. In contrast with V1, V2 lacks an α-helix between the Q1 and KID domains. Both V3 and V4 have lost domain Q1 (Figure 1C). V3 and V5 have been submitted to GenBank under the accession Nos. OL439245 and OL439247, respectively.

### 2.2. Tissue Expression Profile

**Figure 2:**
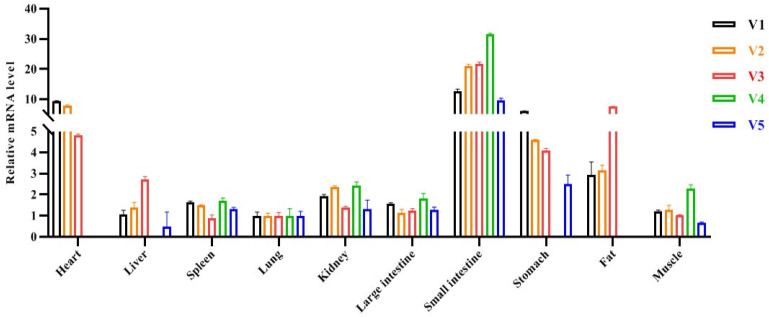
Tissue expression profile of five transcript variants of the porcine *CREB* gene.

Real-time PCR analysis showed that isoforms V1–3 were ubiquitously expressed in all the tissues studied. V4 was not detected in the heart, liver, stomach, and fat tissues. V5 was not found in the heart and fat tissues (Figure 2).

### 2.3. Transcriptional Regulatory Effects of the CREB Variants

To analyze the transcriptional transactivation activity of the in-frame variants, V1–4, dual-luciferase reporter analysis was performed on the basis of bioinformatic analysis. The results demonstrated that there were potential CREB-binding sites in the promoters of the porcine B-cell CLL/lymphoma 2-like protein 2 (BCL2L2) and the poly (A)-binding protein, nuclear 1 (PABPN1) genes.

**Figure 3:**
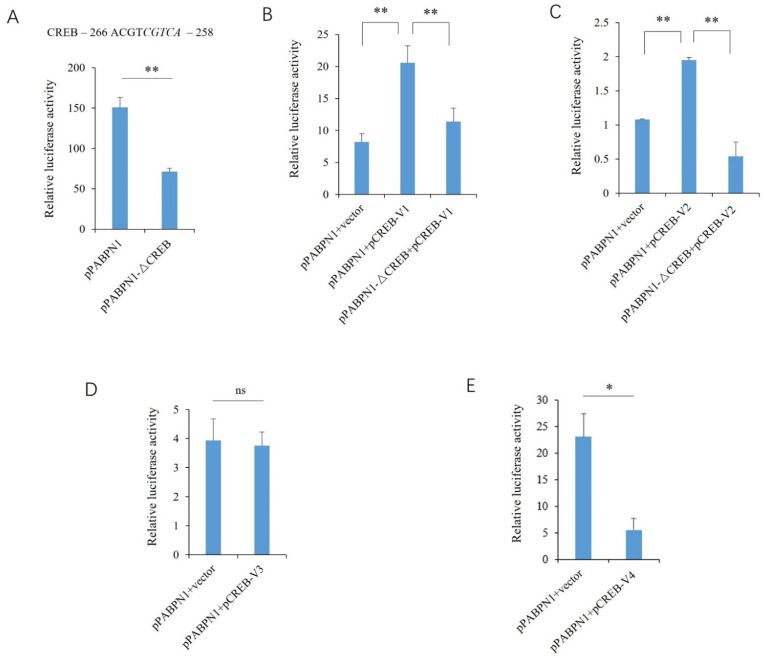
Effects of the transcript variants of CREB on the porcine PABPN1 promoter. (**A**) Effects of CREB motif deletion on promoter activity. The putative binding site is shown above, and the sequence deleted in the mutant-type reporter gene is indicated in italics. (**B**) Effects of V1 overexpression on promoter activity. (**C**) Effects of V2 overexpression on promoter activity. (**D**) Effects of V3 overexpression on promoter activity. (**E**) Effects of V4 overexpression on promoter activity. A Student’s *t*-test was used to compare the differences between the two groups. * and ** indicate that the differences were significant, at *p* < 0.05 and *p* < 0.01 levels, respectively, while ns, not significant, indicates that the differences were not significant (*p* > 0.05).

The reporter gene of PABPN1 has strong promoter activity, as revealed by the luciferase activity analysis. The deletion of the putative cis-element resulted in a significant decrease in the promoter activity (*p* < 0.01) (Figure 3A). The overexpression of CREB V1 or V2 promoted the activity significantly, while no promotive effects of exogenous V1 or V2 were found on the plasmids lacking the binding site of CREB (Figure 3B,C). The overexpression of CREB V3 had no significant effect on the promoter activity of the *PABPN1* gene (*p* > 0.05) (Figure 3D). The ectopic expression of V4 decreased the promoter activity significantly (*p* < 0.05) (Figure 3E).

**Figure 4:**
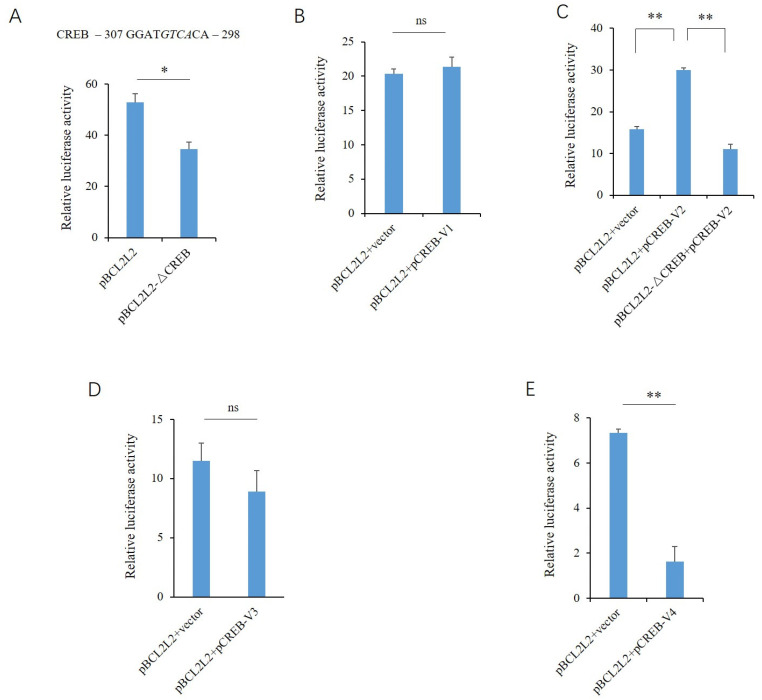
Effects of the transcript variants of CREB on the porcine BCL2L2 promoter. (**A**) Effects of CREB motif deletion on promoter activity. The putative binding site is shown above, and the sequence deleted in the mutant-type reporter gene is indicated in italics. (**B**) Effects of V1 overexpression on promoter activity. (**C**) Effects of V2 overexpression on promoter activity. (**D**) Effects of V3 overexpression on promoter activity. (**E**) Effects of V4 overexpression on promoter activity. A Student’s *t*-test was used to compare the differences between the two groups. * and ** indicate that the differences were significant, at *p* < 0.05 and *p* < 0.01 levels, respectively, while ns, not significant, indicates that the differences were not significant (*p* > 0.05).

The reporter gene of BCL2L2 has strong promoter activity, and the deletion of the putative binding site inhibits the promoter activity significantly (*p* < 0.01) (Figure 4A). The overexpression of CREB V1 had no effect on the luciferase activity, while exogenous V2 increased the promoter activity significantly (*p* < 0.01) (Figure 4B). The deletion of the CREB motif in the reporter gene abolished the effects of exogenous V2 on the promoter activity (Figure 4C). The effects of V3 and V4 on the porcine *BCL2L2* promoter were similar to that on the *PABPN1* promoter. V3 did not significantly affect the promoter activity (Figure 4D), while V4 decreased the activity (Figure 4E).

### 2.4. Effects of the CREB Variants on the Endogenous Expression of PABPN1 and BCL2L2

**Figure 5:**
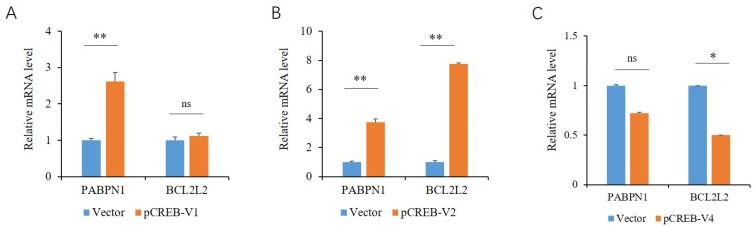
Effects of variants V1 (**A**), V2 (**B**), and V3 (**C**) of CREB on the expression of endogenous *PABPN1* and *BCL2L2* genes. A Student’s *t*-test was used to compare the differences between the two groups. * and ** indicate that the differences were significant, at *p* < 0.05 and *p* < 0.01 levels, respectively, while ns, not significant, indicates that the differences were not significant (*p* > 0.05).

We measured the effects of the CREB transcript variants on the expression of endogenous *PABPN1* and *BCL2L2*. Real-time PCR analysis showed that the overexpression of V1 significantly increased the mRNA level of *PABPN1* in PK-15 cells (*p* < 0.01), while no change was observed in the expression of *BCL2L2* (*p* > 0.05) (Figure 5A). Exogenous V2 increased the expression of both *PABPN1* and *BCL2L2* significantly (*p* < 0.01) (Figure 5B). The overexpression of V4 inhibited the expression of *PABPN1*, but the change was not significant (*p* > 0.05), while it decreased the mRNA level of *BCL2L2* significantly (*p* < 0.05) (Figure 5C).

### 2.5. Comparison of Variants V1 and V2

**Figure 6:**
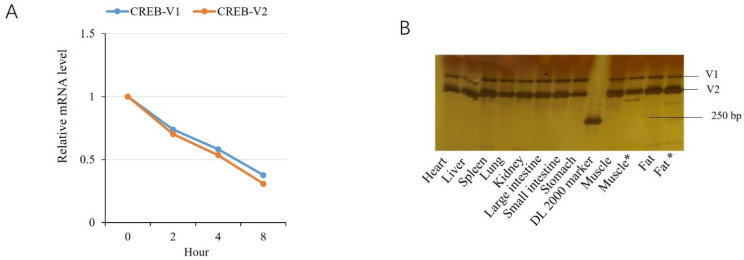
Comparison of isoforms V1 and V2. (**A**) mRNA stability analysis. The mRNA level is shown as the ratio of mRNA remaining to that at time zero. (**B**) Comparison of the mRNA levels in the same tissues. * indicates that the tissues were from 7-month-old pigs, while the other tissues were from 1-month-old pigs.

The above analysis showed that there are differences between the transcriptional regulatory activities in variants V1 and V2, although both increased the transcription of target genes. We studied the mechanisms underlying these differences. Transcripts V1 and V2 are similar in stability, as revealed by half-time analysis (Figure 6A). Competitive RT-PCR was used to compare the expression levels of the two variants in the same tissues, and it revealed that V2 was expressed more abundantly than V1 in all the tissues examined (Figure 6B).

### 2.6. Overview of the RNA-Seq Data

RNA-seq analysis was used to reveal the mechanisms underlying the differential roles of variants V1 and V2 in regulating gene expression. A total of 179.43 million clean read pairs were obtained in nine cDNA libraries, with 19.24, 19.09, and 21.48 million on average for cells transfected with V1, V2, and an empty vector (negative control, NC), respectively, comprising at least 94.3% of Quality 20 (Q20) reads and 86.6% of Q30 reads. More than 93.12% of the clean reads in each sample were mapped to the reference genome (*Sus scrofa* 11.1).

**Figure 7:**
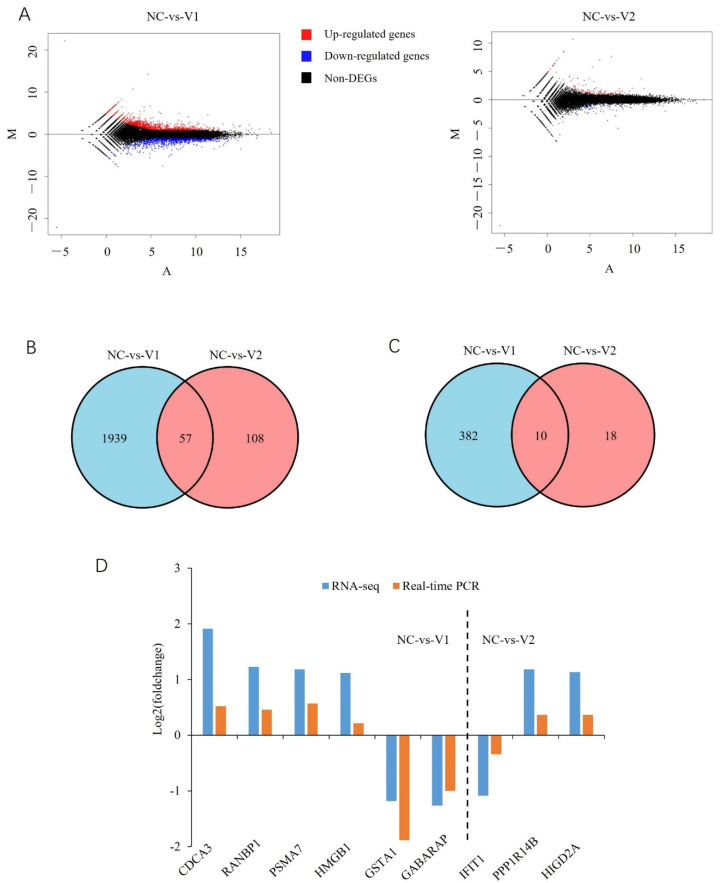
Overview of the RNA-seq data. (**A**) MA plot of the RNA-seq data. (**B**) Venn diagram of DEGs in cells overexpressing V1 and V2. (**C**) Venn diagram of differentially expressed transcription factors in cells overexpressing V1 and V2. (**D**) Validation of the RNA-seq data with real-time quantitative PCR.

When compared to the NC group, there were 1996 differentially expressed genes (DEGs), with 1046 upregulated genes and 950 downregulated genes in cells overexpressing V1. There were 165 DEGs, with 95 upregulated genes and 70 downregulated genes in cells overexpressing V2 (Figure 7A; Table S1A,B). Among the DEGs regulated by V1 and V2, 57 were common (Figure 7B). DEGs specifically regulated by V1 or V2 are shown in Table S1C. A total of 392 and 28 DEGs were identified as TFs in the V1- and V2-treated groups, respectively, of which 10 were common to the two groups (Figure 7C; Table S2). Nine DEGs, six from the NC-vs.-V1 group and three from the NC-vs.-V2 group, were selected to validate the RNA-seq data with real-time PCR analysis, and consistent results were obtained (Figure 7D).

### 2.7. Functional Characterization of the Differentially Expressed Genes

**Figure 8:**
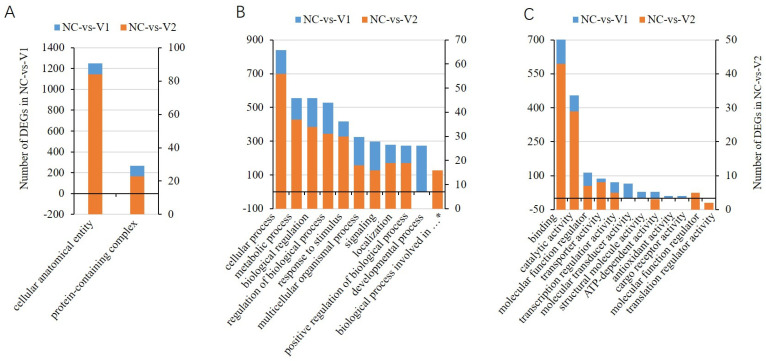
Top GO terms involved in the cellular component (**A**), biological process (**B**), and molecular function (**C**). * Biological process involved in the interspecies interaction between organisms.

Gene Ontology (GO) and Kyoto Encyclopedia of Genes and Genomes (KEGG) analyses were performed on the NC-vs.-V1 and NC-vs.-V2 groups, and the results were compared to reveal the reason for their differential roles in the regulation of gene expression. Two GO terms were enriched in the cellular component category in each group, and there was no difference between the two groups (Figure 8A). Among the top 10 GO terms enriched in the biological pathway category, nine were common to the two groups. The terms developmental process and biological process involved in the interspecies interaction between organisms were specific to NC-vs.-V1 and NC-vs.-V2, respectively. In the molecular function category, a greater number of items differed between the two groups (Figure 8B). A total of six GO terms were differentially enriched in the two groups, of which the molecular transducer activity, structural molecule activity, antioxidant activity, and cargo receptor activity were specific to NC-vs.-V1, while the molecular function regulator and translation regulator activity were specific to NC-vs.-V2 (Figure 8C).

**Figure 9:**
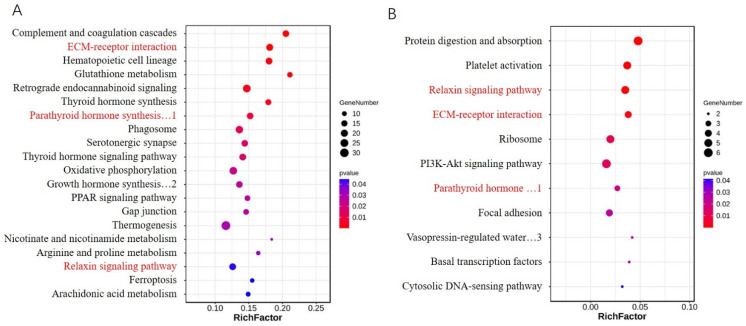
KEGG pathways significantly enriched by DEGs, regulated by V1 (**A**) and V2 (**B**). Pathways shared by the two groups are indicated in red. 1. Parathyroid hormone synthesis, secretion, and action. 2. Growth hormone synthesis, secretion, and action. 3. Vasopressin-regulated water reabsorption.

DEGs were involved in various pathways in both the NC-vs.-V1 and NC-vs.-V2 groups. Pathways enriched in NC-vs.-V1 were very different from those enriched in the NC-vs.-V2 group. The most significantly enriched pathways were arachidonic acid metabolism and ferroptosis in the NC-vs.-V1 group, and the cytosolic DNA-sensing pathway and basal TFs in the NC-vs.-V2 group. Among all the significantly enriched pathways (*p* < 0.05), only three were common to both groups (Figure 9A,B). Both groups contained pathways related to adipogenesis, such as arachidonic acid metabolism and the PPAR signaling pathway in NC-vs.-V1, and the PI3K-Akt signaling pathway and focal adhesion in NC-vs.-V2. This suggests that the two variants regulate fat deposition via different mechanisms.

## 3. Discussion

CREB is a critical TF involved in various physiological processes. It is rich in AS transcripts, some of which play distinctive roles in cell growth and development, while some isoforms, such as α and β in humans and mice, have similar tissue expression profiles and identical domains [21], suggesting that they play the same roles. However, these isoforms may not be redundant in the transcriptome and the proteome, and little effort has been made to analyze the functional differences between them. In this study, AS transcript variants of CREB were characterized in pigs using molecular biology techniques. Through dual-luciferase reporter analysis, four of the isoforms containing in-frame variations were analyzed to compare their transcriptional transactivation activities in PK-15 cells. The mechanisms underlying the differential activities of two canonical isoforms, corresponding to α and β in humans and mice, were analyzed at the genome level. The results help to reveal the role of CREB and to analyze the significance of AS in genes.

Five transcripts of CREB, V1–5, were characterized in pigs, and two, V3 and V5, were identified for the first time. In contrast to V1, isoforms V2–4 are in-frame variations. V2 contains all the same functional domains of CREB as V1, while isoforms V3 and V4 have lost the Q1 domain because AS now occurs in that region. The CREB protein is composed of distinct domains with different functions. N-terminal domains, including Q1, Q2, and KID, function synergistically to induce the transcriptional transactivation of CREB. Q1 functions to induce transcriptional enhancement, and Q2 is essential for inducing CREB-mediated transcription. The C-terminal portion harbors the bZIP domain responsible for the DNA binding and dimerization of CREB. The KID domain, intermediated between Q1 and Q2, can be activated by a series of kinases, such as protein kinase A, which affects the dimerization and DNA binding of CREB [26]. Thus, V3 and V4 have domains that are essential for transcriptional regulation, suggesting that they have transcriptional transactivation activity.

The expression profiles of transcripts V3 and V4 in tissues indicate that they have distinctive roles. V1 and V2, composed of identical domains, are ubiquitously expressed with similar profiles, indicating their identical roles. However, it is impossible to express two isoforms with identical roles simultaneously. To reveal the differences between isoforms V1 and V2 and to verify the potential of V3 and V4 as TFs, dual-luciferase reporter analysis was performed using *PABPN1* and *BCL2L2* as representatives of the target genes. We found that both V1 and V2 can promote the transcription of *PABPN1*, while only V2 has transcriptional regulation activity in the promoter of *BCL2L2*. This indicates that there is a division of labor between V1 and V2 in activating gene expression. V3 has no effect on the promoter activity of the two genes, while V4 inhibits the expression of both *PABPN1* and *BCL2L2*, in contrast to the promoter-activating ability of V1 and V2. Conflicting roles in the regulation of gene expression have been observed in the AS isoforms of the *CREB* gene. It is predominantly a positive regulator of the cAMP-responsive genes, but in the human testis, repressor CREB isoforms have been characterized [27,28], indicating the multiple roles played by the AS of *CREB*.

CREB is a bifunctional transcription activator, mediating both the constitutive and kinase-induced transcription activations of numerous genes in a variety of cell types [24,29,30]. It exerts its effects through Q2, a constitutive activation domain, for activating constitutive transcription, and KID for kinase-inducible activities. Although the Q2 domain is crucial for constitutive activation, both Q1 and Q2 are important for basal activation [30]. In contrast to V2, a positive regulator of gene expression, V4 is formed by exon-skipping of exon 4, coding for the Q1 domain in CDS. Therefore, V2 and V4 are produced by exon 4 splicing in and out, respectively, and they are differentiated from one another by Q1 inclusion or deletion. A similar phenomenon was also observed in the cAMP-responsive element modulator (CREM), another member of the CREB family that functions as a transcriptional activator. CREM can produce an isoform that explicitly antagonizes the transcriptional activation. Exon 9 of CREM coding for the Q domain is alternatively spliced. Its inclusion or deletion in the CREM mRNA results in a switch of the protein from functioning as an activator to acting as a repressor during spermatogenesis [31].

Q1 is responsible for transcriptional enhancement. It is unclear why its deletion in V4 results in antagonistic activity. Possibly, the deletion causes conformational alteration of the polypeptide, which renders it unable to form productive interactions with the proteins involved in the basal transcription complex. Additionally, V4 has a bZIP domain responsible for the DNA binding at the C-terminal, and, thus, it might compete with the activator CREB isoforms to bind to the CRE motif and inhibit the cAMP-stimulated gene expression. Nevertheless, V4 can function as a TF, and may account for the tissue-specific repression of cAMP-regulated genes because it is not expressed in all tissues, as revealed by real-time PCR.

Isoforms V1 and V2 have identical domains, similar stability, and tissue expression profiles, except for a higher mRNA level in V2 than in V1 in the same tissues. However, V1 and V2 showed differences in their transcriptional transactivation of genes. To reveal the underlying mechanisms, RNA-seq analysis was performed and the DEGs regulated by V1 and V2 were identified. There are only a few common genes significantly regulated by both V1 and V2 (absolute log2-foldchange > 1 and *p* < 0.05), and ectopic V1 altered the expression of many more genes than V2. These data indicate that there is a great difference between their regulations of gene expression. Specific GO terms and KEGG pathways enriched by DEGs were also identified in each group. There was a small difference between the GO terms enriched by both groups, especially in the CC and BP categories, while in the KEGG pathways, a greater difference was observed. Among the top 10 pathways enriched, only three were common to both groups, which indicates that different pathways might be the main reason for the differential roles of V1 and V2.

## 4. Materials and Methods

### 4.1. Animals, Tissues, and cDNA Synthesis

One-month-old and seven-month-old Min pigs were sampled from the Institute of Animal Husbandry, Heilongjiang Academy of Agricultural Sciences, Harbin, China, each with three replicates. Animal handling was carried out in accordance with the protocols approved by the Animal Care and Use Committee at Northeast Agricultural University (Harbin, China). Tissues, including those from the heart, liver, lungs, spleen, kidneys, stomach, large intestine, small intestine, fat, and muscles were collected immediately after the pigs were slaughtered, and these tissues were snap-frozen in liquid nitrogen. RNA was isolated with TRIzol reagent (Invitrogen, Carlsbad, CA, USA), and reverse transcription (RT) was performed, as previously described, to synthesize cDNA [32].

### 4.2. cDNA Amplification and Sequence Analysis

To characterize the AS events in CDS, a pair of primers were designed according to the porcine *CREB* (pCREB) mRNA deposited in GenBank (NM_001361427). PCR was carried out in a final volume of 25 μL containing 1 U exTaq DNA polymerase (Takara, Dalian, China), 1× PCR buffer, 0.2 μM of each primer, 200 μM of each dNTP (Takara, Dalian, China), and 1 μL of cDNA obtained from muscle tissues. The thermal cycling parameters were as follows: 94 °C for 5 min, followed by 30 cycles at 94 °C for 30 s, 59 °C for 30 s, 72 °C for 1 min, and a final extension at 72 °C for 7 min. All primers used in this study are listed in Table S3.

PCR products were inserted into the pMD18-T vector (Takara) for sequencing by Beijing Genomic Institute (BGI, Beijing, China). The resultant sequences were aligned using SnapGene (v5.2.4). The genomic structure was characterized using the Blat program (http://genome.ucsc.edu/cgi-bin/hgBlat, accessed on 3 March 2022).

### 4.3. Plasmids

To analyze the transcriptional transactivation potential of the AS variants of pCREB, the CDSs of isoforms encoding polypeptides with functional domains were amplified with primer pair A, containing *Eco*RI and *Xho*I sites in the 5′ end, and subcloned into a pCMV-HA vector to construct eukaryotic expression plasmids. Luciferase reporter genes of porcine BCL2L2 and PABPN1 were constructed with pGL3-basic as the backbone at the *Kpn*I and *Hin*dIII sites. The potential of the upstream sequences of the *BCL2L2* or *PABPN1* genes as promoters and the putative binding sites of CREB were first analyzed with Promoter 2.0, Alibaba 2.1, and Jaspar programs. Then, sequences spanning from −1102 to +54 nucleotides (nt) of *BCL2L2* and −791 to −190 nt of *PABPN1* were amplified from genomic DNA and then inserted into the pGL3-basic. The first nucleotide of the start codon was assigned as +1. Additionally, the predicted CRE elements were deleted from the *BCL2L2* and *PABPN1* reporter genes using an overlapped extension PCR method, as described previously [32].

### 4.4. Dual-Luciferase Reporter Assay

A dual-luciferase reporter assay was performed, as described previously [32]. Briefly, each reporter gene constructed was transfected alone or together with plasmids overexpressing *pCREB* into PK-15 cells using Lipofectamine 2000 (Invitrogen). The *Renilla* luciferase reporter gene was used as the reference to avoid differences in transfection efficiency among the groups. At 48 h after transfection, the cells were collected and the luciferase activity was measured with the Dual-Glo Luciferase Assay System (Promega, Madison, WI, USA). The relative luciferase activity was calculated as the ratio of firefly activity to that of *Renilla*. A Student’s *t-*test was used to compare the differences between the experiment and control groups. Each experiment was independently repeated three times, each with triplicates. Data are presented as mean ± standard deviation.

### 4.5. Competitive RT-PCR

Isoforms V1 and V2 were amplified simultaneously using the competitive RT-PCR method to compare the expression levels in the same tissue. A pair of primers, complementary to the common sequences of both variants, was used. The PCR volume and reaction protocols were the same as those described above. The products were visualized using 12% polyacrylamide gel electrophoresis.

### 4.6. Library Preparation and Sequencing

Plasmids overexpressing *pCREB* were transiently transfected into PK-15 cells with Lipofectamine 2000 reagent (Invitrogen) according to the manufacturer’s protocol. Cells transfected with an empty vector were used as the control. At 48 h after transfection, cells were collected, and RNA was isolated with TRIzol reagent (Invitrogen) to construct paired-end RNA-seq libraries. Three independent experiments were performed. A total of six libraries were prepared from the experimental and control groups, each with three replicates. The libraries were prepared and sequenced on a NovaSeq 6000 sequence analyzer (Illumina, San Diego, CA, USA) by the Frasergen company (Wuhan, China), as described previously [33].

### 4.7. RNA-Seq Data Analysis

The raw reads were filtered with SOAPnuke (v2.1.0) to obtain clean reads. The clean reads were aligned to the pig reference genome (Sscrofa 11.1) with the HISAT2 (v2.1.0) program [34]. Bowtie 2 (V2.3.5) [35] was used to map the reads to the ENSEMBLE transcriptome (*Sscrofa 11.1*). The transcript assembly and quantification in fragments per kilobase million (FPKM) were performed with Cufflinks (v2.2.1) [36]. The threshold for expression was set at FPKM > 0.1 in at least one sample, as used previously [33]. The gene expression levels were compared between groups with DESeq2 (v1.22.2) [37], and DEGs were identified according to the criteria of absolute Log2-foldchange > 1 and *p* < 0.05. GO and KEGG enrichment analyses of DEGs were carried out with the hypergeometric distribution method and KOBAS tool (v3.0) [38], respectively. KEGG pathways with a *p*-value < 0.05 were considered as significantly enriched. Hmmscan (v3.0) [39] was used to predict the TFs.

### 4.8. Real-Time Quantitative PCR

Real-time quantitative (q) PCR was performed as described previously with β-actin as the reference [32]. Each of the AS transcripts of the *pCREB* gene were amplified specifically to measure the mRNA levels in the tissues and cells. The cells overexpressing *pCREB* were collected at 48 h after transfection for RNA isolation. To measure the mRNA stability of isoforms V1 and V2, PK-15 cells were treated with 5 µg/mL actinomycin D (ActD, Biotopped, Beijing, China) and collected for RNA isolation at each time point described in the Results section. The relative mRNA levels were determined as the ratio of mRNA remaining to that at time zero. For the validation of the RNA-seq data, nine DGEs were selected, and qPCR was performed with cDNA obtained from cells treated identically to those used for RNA-seq analysis. Data are shown as mean ± standard deviation.

## 5. Conclusions

A total of five AS isoforms of porcine CREB, named V1–5, were cloned. Two of these isoforms, V3 and V5, were identified for the first time. These data indicate that the *CREB* gene is rich in AS. Isoforms V1–3 were ubiquitously expressed in all the tissues sampled, while V4 and V5 were not. V1 and V2 contain all the functional domains of CREB and have similar stability and tissue expression profiles, suggesting that they have the same functional roles. Through dual-luciferase reporter analysis on the promoters of *PABNP1* and *BCL2L2* genes containing the putative CRE motif, we found that V1 and V2 both function as activators, but their roles are gene-specific. To analyze the mechanisms underlying these differences, genes, GO terms, and KEGG pathways specifically regulated by V1 or V2 were identified by RNA-seq combined with bioinformatic methods. We found that V3 had no effect on the promoter activities of *PABNP1* and *BCL2L2*, while V4 acted as a repressor in regulating gene expression. These results demonstrate that CREB isoforms play multiple roles.

## Data Availability

The cDNA sequences of CREB have been deposited in GenBank data and the accepted Nos. are provided in the manuscript. All the relevant data are provided along with the manuscript as Supplementary Files.

## Supplementary Materials

The following supporting information can be downloaded at: https://www.mdpi.com/article/10.3390/genes13081304/s1, Table S1: Differentially expressed genes identified, Table S2: Differentially expressed transcription factors identified, Table S3: Primers used in this study.
