# Supplementary material for: Alternative Splicing Isoforms of Porcine CREB Are Differentially Involved in Transcriptional Transactivation
Source: Genes (Basel). 2022 Jul 22;13(8):1304. doi: 10.3390/genes13081304 (PMC9331954; doi:10.3390/genes13081304)
Supplement: Supplementary file 1 [file genes-13-01304-s001.zip › Table S3 primers used in this study.pdf]

Table S1. Primers used in this study

Sequences (5'-3')	Purpose
	CDS amplification
F: ATGACCATGGAATCTGGAGCAGACAAC<br>R: TTAATCTGATTTGTGGCAGTAAAGGTCC	
	Competitive RT-PCR
F: GCCACAGATTGCCACATTAGC<br>R: CTGACTCCTGTGAATCTTCGCTT	
	Real-time PCR for CREB measurement
F: CTCCGGAACTCAGATTTCAACTA<br>R: TCCTGGTGCGTCAGAAGATAA	V1
F: CAAGTCCAGACAGTTCAGATTTCAA<br>R: ACTCCTGGTGCGTCAGAAGATAA	V2
F: CTCACGCAACATCAGTTATTCAGTC<br>R: the same as V2R	V3
F: TGCCACATTAGCCCAGATTTCAAC<br>R: CACTCCTGGTGCGTCAGAAGATAA	V4
F: GCTATGAAAGACAGCAATGAACAAA<br>R: the same as V1R	V5
	Promoter amplification
F: GGTACCGCACGGTTAGAACTTGTGACTGG<br>R: AAGCTTCAATGGACTCCTAGCTCGCC	PABPN1
F: GGTACCGTCTCCAGCCACCCGCTTTCTTC<br>R: CCCAAGCTTCACAAAGTCTGCCACTAGAGCCCGT	BCL2L2
	Deletion of CREB motif
F: GAGAAGCAAGACAGCGTGGCGG<br>R: CCGCCACGCTGTCTTGCTTCTC	PABPN1
F: CTTGTAGGGATCAGCCCTGCTGTTG<br>R: CAGCAGGGCTGATCCCTACAAGCC	BCL2L2
	Real-time PCR for validation of RNA-seq
F: GCTTCGGGAGTTGAGGAT<br>R: GCAGGTTTGGCTGTGGAG	CDCA3
F: GCACGGATAGTCATCAACAGG<br>R: GTCAAAGTCAAAACCCACGA	PSMA7
F: TAAGAAGTGCTCAGAAAGGTG<br>R: GCGGTGTTATTCCACATCTC	HMGB1
F: CGGTGATAGTAGAAAAGGCTC<br>R: CACTGGTGGGTGGAATGAC	GABARAP
F: GCCGAGGCAGAATGGAGTGT<br>R: GGTGGCGATGTAGTTGAGGA	GSTA1
F: CAGTTTGAGCCGATAGTTTCT<br>R: GGCGTGATGTAGTGATTGG	RANBP1
F: AATCCGCCTGAATCCCA	IFIT1

R: GCAGCCTCATTTTGACCAT	
F: ACGCCAAGGGAAGGTCA	PPP1R14B
R: CAGGAGTTCATCCACATCAATC	
F: AAGTTTATTCGCAAGACCCG	HIGD2A
R: AGACCCACCAAGATGGCTAC	

F, forward; R, reverse; restriction sites were underlined.
